# Socio-demographic Predictors of Hospitalization Duration Among Patients with Borderline Personality Disorder

**DOI:** 10.1007/s10488-024-01388-w

**Published:** 2024-05-30

**Authors:** Amit Yaniv-Rosenfeld, Elizaveta Savchenko, Maya Netzer, Amir Elalouf, Uri Nitzan

**Affiliations:** 1 Shalvata Mental Health Care Center, Hod Hasharon, Israel; 2 Faculty of Medicine, Tel-Aviv University, Tel-Aviv, Israel; 3 Department of Management, Bar-Ilan University, Ramat-Gan, Israel; 4 Department of Mathematics, Ariel University, Ariel, Israel; 5 Department of Information Science, Bar-Ilan University, Ramat-Gan, Israel

**Keywords:** Borderline personality disorder, Hospitalization duration, Socio-demographic predictors

## Abstract

Borderline personality disorder (BPD) is a complex psychopathology associated with high service utilization rates. In turn, the hospitalization of BPD patients is a controversial challenge for mental health professionals. Prior literature has identified certain socio-demographic factors as linked to an increased risk of BPD. In this study, we examined the possible connection between these socio-demographic factors and hospitalization duration. We analyzed 1077 hospitalization records of 200 BPD-diagnosed patients. Patients’ gender, age, education level, employment and marital statuses, and living arrangement were statistically significantly linked with hospitalization duration. Specifically, female gender, age twenty or below, no high-school diploma (or, to a lesser extent, a diploma with no academic education), unemployment status and/or patients who live with parents are strongly associated with longer hospitalizations compared to male gender, older patients, more educated, married/divorced status and/or those who do not live with their parents. Additionally, the results point to a weak, albeit statistically significant, temporal pattern with more advanced hospitalizations generally aligning with the duration of their preceding ones, while being slightly shorter. In order to prevent potentially unnecessary prolonged and regressive hospitalizations, an estimation of the expected hospitalization duration should be explicitly considered when setting hospitalization goals and plans.

**Supplementary Information:**

The online version contains supplementary material available at 10.1007/s10488-024-01388-w.

## Introduction

Borderline personality disorder (BPD) is a complex psychopathology often characterized by intense emotional instability, impulsive behavior, and unstable relationships. BPD affects approximately 0.7% to 2.7% of the US adult population (Leichsenring et al., 2023), yet higher prevalence rates are commonly reported in primary care (6%), outpatient psychiatric services (11–12%), and inpatient psychiatric hospitalization (22%) (Ellison et al., 2018; Zimmerman & Becker, 2022). BPD is typically associated with one of the highest service utilization rates (Comtois et al., 2003) and substantial burnout rates among mental health professionals (Finamore et al., 2020; Perseius et al., 2007).

From a socio-demographic perspective, there is no single socio-demographic factor that is consistently associated with BPD; individuals from all ages and backgrounds can suffer from it National Institute of Mental Health (2023). Nevertheless, certain socio-demographic factors are known to be linked with an increased risk of being diagnosed with BPD. Notably, BPD is typically diagnosed in early adulthood and significantly more often in females than in males (American Psychiatric Association, 2013). In addition, individuals from lower socio-economic backgrounds are generally at higher risk of developing BPD as they also suffer from a higher incidence of adverse life events such as childhood trauma, abuse, neglect, poverty, etc. Estric et al. (2022). Marital and employment statuses, as well as one’s living arrangement, were also found to be linked with BPD; only 16% of patients suffering from BPD are married or living with a partner and only 35% had regular work or school performance (Zanarini et al., 2015). Unfortunately, to the best of our knowledge, any potential link between these socio-demographics and one’s hospitalization duration has yet to be examined.

Inpatient treatment of BPD patients in general, and its duration in particular, are major areas of contention among mental health clinicians (Bateman & Fonagy, 1999, 2001). One key consideration is the “revolving door” phenomenon, a pattern of multiple frequent hospitalizations, which is unfortunately common for BPD patients primarily due to their impulsivity, emotional dysregulation, and unstable interpersonal relationships (Fonseca Barbosa & Gama Marques, 2023; Morlino et al., 2011). When hospitalization is needed, the American Psychiatric Association suggests that an extended hospitalization among BPD patients should only be moderated and considered under specific circumstances; for example, those with persistent and severe suicidality or comorbid substance abuse or dependence (Oldham et al., 2010). Accordingly, inpatient treatment duration for BPD has been gradually reduced over the years from months to weeks and even days. This reduction is often associated with increased utilization of pharmacotherapy and the discharge of unstable and suicidal patients (Biskin & Paris, 2012; Lake et al., 1993; Gunderson, 2009). Possibly, the challenging counter-transference and negative attitudes of staff members might also contribute to the shortening of BPD hospitalization duration (Fallon, 2003). Despite the controversy regarding the efficacy of hospitalizations, the treatment of many BPD inpatients, especially those who face high levels of distress or pose a significant risk to themselves or others, still necessitate a prolonged hospitalization period (Oldham, 2006; Paris, 2002). During such an hospitalization period, BPD patients typically undergo a multifaceted treatment regimen encompassing crisis intervention, medication management, individualized support therapy, group therapy, and/or additional interventions that are tailored to the severity of symptoms, individual patient preferences, resource availability, and other pertinent considerations (Storebø et al., 2020). Unfortunately, the extended hospitalizations of BPD patients are, at times, a substantial burden for mental health institutions—both economically and in terms of staff burnout rates. Thus, identifying BPD patients who are prone to prolonged hospitalization could be highly valuable for deriving more suitable treatment plans and goals at admission to the hospital.

In the broader context of mental healthcare research, previous studies have examined the potential connection between socio-demographic factors and the duration of patient hospitalizations. Central to these is the recognition that hospitalization resources are limited, prompting the need for more effective goals, plans, and treatments in order to mitigate unnecessarily prolonged stays (i.e., deinstitutionalization) (Bassuk & Gerson, 2022). Unfortunately, in our context, existing understanding of the matter is limited in two major ways: First, over the years, most of the studies have been conducted in the United States (US), raising concerns as to its consistency outside the US (Tulloch et al., 2011). Specifically, we are unaware of any such study that was conducted in Israel. Second, to our knowledge, prior studies have not deliberately focused on BPD, a highly prevalent and challenging condition in in-patient settings as discussed before. In the present study, our primary objective is to examine the extent to which a BPD patient’s expected hospitalization duration at intake can be estimated by his/her socio-demographic characteristics (gender, age, education level, employment status, marital status, and living arrangement) and previous hospitalizations (number and duration). Our hypothesis is that the socio-demographic factors previously linked with BPD diagnosis will also prove useful in explaining the hospitalization duration of BPD patients.

## Materials and Methods

Methodologically, we followed a three-phased workflow. First, we acquired and annotated contextual data. Then, we analyzed that data in two complementary ways: first by using standard correlation testing and between-groups statistical comparisons, and then using regression analysis.

### Data

For data acquisition, we retrieved and analyzed records from the Shalvata Mental Healthcare Centre (Israel). Shalvata Mental Healthcare Centre serves a total population of roughly 600,000 people and operates 4 adult inpatient departments. Thirty percent of the inpatients at the Shalvata Mental Health Center are diagnosed with BPD based on the DSM-V (American Psychiatric Association, 2013). This estimation has been rather consistent over the last few years.

We randomly selected, and retrospectively analyzed the anonymized files of 200 adult patients with a diagnosis of BPD who were admitted at least once to the hospital between 2018 and 2021. All recorded hospitalizations of these patients, 1077 in total, were anonymously retrieved and manually analyzed. Day visits and scheduled hospitalizations, such as routine follow-ups, were excluded from consideration. The sample considered in this study is similar in size to previous BPD research (Zittel Conklin & Westen, 2005; Zlotnick et al., 2002).

### Measures

Each hospitalization was represented by the following patient’s socio-demographic characteristics at intake: Gender (Male, Female, Other), Age (in years), Education level (No high-school diploma, Partial high-school diploma, High-school diploma, Academic), Employment status (Unemployed, Part-time, Full), Marital status (Single, Married, Divorced, Widower), Kids (yes, no), and Living arrangement [Alone, Partner/Spouse, Parents, Extended Family, Roomate(s)]. In addition, each hospitalization was designated by its index, indicating the number of previous hospitalizations for that patient plus one (i.e., the ordinal number of the hospitalization) and its duration (in days). Overall, each hospitalization was characterized by nine variables; seven of them were socio-demographic and two were connected to the hospitalization.

Importantly, these variables, except for the last one (hospitalization duration), are normally readily available at patient intake. Unfortunately, while some features were explicitly coded in the patient’s electronic health record (such as gender and age), other features such as education level, employment status, and living arrangement were not. As such, one of the co-authors performed a manual review of the hospitalization records and annotated them. These annotations were later validated by two independent reviewers prior to the data analysis. No disagreements were encountered. All records in our study sample included the full set of nine variables mentioned above.

### Statistical Approaches

Descriptive statistics are reported in a standard form with continuous variables presented as mean standard deviation ($SD$) and categorical variables are presented as percentages. Correlation testing was performed using both the Pearson and Spearman tests (Freedman et al., 2007). Between-group comparisons were performed using either a Student’s t-test or an ANalysis Of VAriance (ANOVA) with post-hoc Tukey HSD testing. Regression analysis was performed using a linear model and the mean least squares method (Draper & Smith, 1998). We used SPSS version 24 for our analysis. The p-value for statistical significance was set at $p \le 0.05$.

## Results

### Descriptive Statistics

Overall, 1077 hospitalization records are present in our sample, originating from 200 BPD-diagnosed patients, resulting in an average of $5.36\pm 7.20$ hospitalization per patient. The sample includes 84.5% (169) females, and 15.5% (31) males (0 classified as other). The patients’ age at the time of hospitalization is $36.8\pm 9.59$. Considering the patients’ education, $32.0\%$ (64) did not have any high-school diploma accreditation while 19.0% (38) had partially completed a high-school diploma, 30.0% (60) graduated from high-school, and 19.0% (38) acquired an academic degree. At the time of intake, 61.0% of the hospitalization (657) were of single patients while the remaining 13.9% (150) and 25.1% (270) were of married and divorced patients, respectively (none of widowers). Similarly, 56.1% of the hospitalizations (604) are of patients who have children. In addition, 69.6% of the hospitalizations (750) are of unemployed patients, 3.7% (40) of part-time employed patients, and 26.6% (287) of full-time employed patients. In turn, 29.2% of these hospitalizations (315) were of patients who live alone, 8.1% (87) of patients living with a spouse or partner, 1.2% (14) with their parents, 53.6% (577) with their extended family and 7.8% (84) with a roommate(s).

The number of hospitalizations per patient varied significantly between 1 and 40 (with an average of $5.36\pm 7.20$). As can be observed from Fig. 2 (provided as supplementary material), most patients in our sample had less than five hospitalizations ($\sim 65\%$) while very few had more than 20 hospitalizations ($\sim 5\%$). The average hospitalization duration is $30.507 \pm 68.620$ days.

### Basic Analysis

We start by examining the correlations between the hospitalization index and its duration as illustrated in Fig. 1 (provided as supplementary material). The results point to a very weak, albeit statistically significant, correlation with former hospitalizations being associated with a slightly longer duration compared to later ones with a Person’s correction of $r = 0.069, \; p < 0.05$ and a Spearman’s correlation of $r = 0.096, \; p < 0.005$. Similarly, the results suggest that a patient’s given hospitalization duration is correlated with that patient’s subsequent hospitalization duration with Pearson and Spearman correlations of $r = 0.121, \; p < 0.05$ and $r = 0.259, \; p < 0.001$, respectively. Combined, these results point to a temporal pattern where more advanced hospitalizations generally align with the duration of their preceding ones, while being slightly shorter.

Next, we consider each examined socio-demographic feature separately and perform between-group statistical comparisons. Starting with gender, hospitalizations of female patients are significantly longer compared to hospitalizations of male patients with an average of $34.67 \pm 61.52$ days compared to $20.35 \pm 47.15$ days, $p < 0.01$. In addition, different age groups demonstrate statistically significant differences with the youngest age group (18–20 years old) being associated with the longest hospitalizations ($105\pm 107$ days), which are about three times longer than the average duration of any other age group, at $p < 0.01$. All other patient age groups do not demonstrate statistically significant differences in pairwise testing. Furthermore, hospitalizations of unemployed patients are significantly longer ($36.07 \pm 68.44$ days) compared to those of either fully employed ($24.000 \pm 32.619$ days) or partially employed patients ($19.00 \pm 13.88$ days), both at $p < 0.05$. In addition, hospitalizations of patients who live with their parents ($158\pm 6.11$) seem to be almost five times longer compared to those of patients who live with their extended families ($32.39\pm 66.08$ days), with the difference being statistically significant at $p < 0.001$. In this context, it is important to note that we did not detect any significant connection between age and living arrangement as shown in the heatmap provided in the supplementary material. All other patients’ living arrangements do not differ significantly in this respect. No statistically significant differences are found across education levels, marital status, and parenthood status. Table 1 summarizes the results.

**Table 1:** Hospitalization duration differences based on the examined socio-demographic features

Feature	Value	Duration	p
Gender	Female	$34.675 \pm 61.522$	$<0.01$
	Male	$20.346 \pm 47.150$	
Age	18–20	$105.000 \pm 107.000$	$<0.01$
	20–30	$30.482 \pm 47.887$	
	31–40	$27.844 \pm 53.680$	
	41–50	$36.930 \pm 67.811$	
	51–60	$17.667 \pm 15.995$	
	61+	$21.000 \pm 28.284$	
Education level	No high-school diploma	$27.459 \pm 47.534$	0.186
	Partial high-school diploma	$25.639 \pm 63.909$	
	High-school diploma	$39.226 \pm 64.378$	
	Academic	$31.980 \pm 59.376$	
Marital status	Single	$33.673 \pm 65.594$	0.365
	Married	$29.732 \pm 49.751$	
	Divorced	$25.510 \pm 40.065$	
Children	Yes	$28.158 \pm 45.526$	0.392
	No	$32.681 \pm 64.090$	
Employment status	Unemployed	$36.074 \pm 68.440$	0.012
	Part-time	$19.000 \pm 13.879$	
	Full-time	$24.000 \pm 32.619$	
Living arrangement	Alone	$29.284 \pm 42.718$	0.025
	Spouse/partner	$17.700 \pm 16.414$	
	Parents	$158.000 \pm 6.111$	
	Extend family	$32.388 \pm 66.081$	
	Roommate(s)	$12.500 \pm 5.500$	

### Regression Analysis

Next, we implemented the following linear regression model to the data. We considered the hospitalization duration as the dependent variable and the socio-demographic features outlined in section “Methods and Materials” as the independent variables as outlined below.

1 $$\begin{aligned} \text {Duration}= & {} \alpha _0 + \alpha _{1} \text { Female} + \alpha _{2} \text { Age} + \alpha _{3} \text { Partial diploma} \nonumber \\{} & {} + \alpha _{4} \text { Diploma} + \alpha _{5} \text { Academic} + \nonumber \\{} & {} \alpha _{6} \text { Married} + \alpha _{7} \text { Divorced} + \alpha _{8} \text { Children} \nonumber \\{} & {} + \alpha _{9} \text { Partially employed} + \alpha _{10} \text { Employed} + \nonumber \\{} & {} \alpha _{11} \text { Partner} + \alpha _{12} \text { Parents} + \alpha _{13} \text { Extended family} \nonumber \\{} & {} + \alpha _{14} \text { Roomate(s)} + \alpha _{15} \text { Visit index} \end{aligned}$$

The obtained coefficients and their significance are reported in Table 2. Specifically, the model seems to suggest that longer hospitalizations are expected among females (by 30.44 days), and those who live with their parents (by 72.6 days) compared to males, and those who live alone, respectively. In addition, some socio-demographic characteristics seem to indicate a shorter hospitalization duration. Particularly, married patients (by 15.14 days), those who are partially or fully employed (by 15.1 and 46.03 days, respectively), live with a spouse or partner (by 34.56 days), and had more previous hospitalizations (by 1.48 days per previous hospitalization) seem to present shorter hospitalization duration compared to patients who are single, unemployed, living alone or with fewer prior hospitalizations. Overall, approximately 9% of variance in hospitalization duration can be explained by the patient’s socio-demographics $(R^2=0.089).$

**Table 2:** Regression coefficients, their significance, and confidence intervals

Parameter	Symbol	Value	p-value	Confidence interval
	$\alpha _{0}$	59.89	0.045	–
Gender (female)	$\alpha _1$	30.44	0.002	[11.45, 49.43]
Age	$\alpha _2$	$-$8.39	0.403	[$-$28.22, 11.44]
Partial diploma	$\alpha _3$	$-$39.30	0.110	[$-$87.68, 9.07]
Diploma	$\alpha _4$	$-$3.46	0.865	[$-$42.84, 47.77]
Academic	$\alpha _5$	22.97	0.307	[$-$21.43, 67.36]
Married	$\alpha _{6}$	$-$15.14	0.034	[$-$20.09, $-$7.13]
Divorced	$\alpha _{7}$	$-$16.83	0.608	[$-$21.83, 9.17]
Children	$\alpha _{8}$	0.25	0.965	[$-$11.31, 11.82]
Partially employed	$\alpha _{9}$	$-$15.10	0.038	[$-$47.71, $-$7.50]
Employed	$\alpha _{10}$	$-$46.03	0.021	[$-$77.85, $-$14.21]
Partner	$\alpha _{11}$	$-$34.56	0.043	[$-$114.59, $-$5.46]
Parents	$\alpha _{12}$	72.60	0.019	[37.52, 182.72]
Extended family	$\alpha _{13}$	3.32	0.849	[$-$31.06, 37.70]
Roomate	$\alpha _{14}$	$-$22.94	0.579	[$-$104.71, 58.83]
Visit index	$\alpha _{15}$	$-$1.48	0.047	[$-$2.53, $-$0.43]

## Discussion

Variability in hospitalization duration among BPD patients can be partly explained by their socio-demographic characteristics. Most notably, the combination of the above bivariate analysis and regression analysis points to the female gender, young age, unemployment, and living with one’s parents as linked with longer hospitalizations compared to the male gender, partial/full employment, and other living arrangements, alone and/or combined.

Historically, BPD has been associated more frequently with female gender (Wilkinson-Ryan & Westen, 2000). However, BPD is now recognized as affecting both genders more evenly than previously believed (Ryden et al., 2008; Gunderson & Kolb, 1978). Nonetheless, some gender-based presentation differences, help-seeking behaviors, and comorbidity, among others, may partially explain the observed gender discrepancies. Specifically, prior literature has pointed out that women with BPD may be more likely to exhibit emotional dysregulation characterized by intense and rapidly changing emotions as well as more internalizing behaviors (e.g., self-harm), whereas men may struggle more with externalizing behaviors, such as anger, aggression, and risk-taking (Maccoby & Jacklin, 1980). As a result, these presentation differences may influence the selection of treatment strategies and their effectiveness. Similarly, it is well established that women tend to pursue mental health support more than men (Wilkinson-Ryan & Westen, 2000). Thus, it may be the case that female patients agree to, or even request, longer hospitalization duration compared to their male counterparts. In addition, men with BPD have higher rates of co-occurring substance abuse and may be more prone to antisocial traits, which could impact treatment planning and administration as well Harkness and Lilienfeld (1997). Considering one’s employment status, it is important to recall that the relationship between any mental health condition and employment is complex and can be influenced by a wide gamut of factors, including social support, access to mental health treatment, coping strategies, and economic circumstances (Bhavsar & Bhugra, 2008). Nevertheless, it is reasonable to expect employed BPD patients to present higher functioning levels and have better symptom management more often than unemployed patients. For example, in order to maintain steady employment, one is usually required to maintain adequate interpersonal skills and be able to properly focus on something other than emotional distress, properties that many BPD patients find very challenging. Similarly to one’s employment status, the relationship between any mental health condition and living arrangement may be associated with one’s daily functioning and overall well-being (Bhavsar & Bhugra, 2008). In our setting, it may be the case that patients who live with their parents cannot properly maintain any other, more independent, living arrangement due to their condition and symptoms (Bartsch et al., 2016; Boucher et al., 2017). In a complementary fashion, these patients may also opt for longer hospitalizations in order to avoid the difficulties associated with living with their parents.

Contextualizing the above results within the wider scope of psychiatric hospitalization duration, a few notable similarities and disparities from existing findings seem to emerge. First, our results seem to align with the prevalent reporting of longer hospitalizations for females (Hodgson et al., 2000; Tulloch et al., 2011), unemployed (Gopalakrishna et al., 2015; Masters et al., 2014) and singles (Douzenis et al., 2012; Noohi et al., 2020) among the general psychiatric inpatient population. Considering age, prior literature has presented equivocal results. Older age was found to be associated with increased hospitalization duration in some studies (Fong et al., 2010; Hodgson et al., 2000; Tulloch et al., 2011) whereas other studies associate older age with shorter hospitalizations (Chung et al., 2013; Peiró et al., 2004; Stevens et al., 2001). Our results seem to align with the latter option, suggesting that at least for the young adult BPD patients (18–20 years old), hospitalization duration is significantly longer. Considering one’s living arrangement, prior studies have primarily considered living alone as a social disadvantage (i.e., lower social and family support) associated with longer hospitalization duration (Dimitri et al., 2018; Parmar et al., 2021). Our results do not fully align with this point of view and seem to suggest that, at least for BPD patients, living alone is an indicator of shorter hospitalization duration compared to those living with their parents and an indicator of longer hospitalization duration compared to those living with a spouse or partner. As such, in our context, living alone may be viewed as a sign of independence and mental strength in a BPD patient yet, at the same time, it may also be a potential indicator of unstable social and romantic relationships.

It is important to note that the studies mentioned above refer to the general psychiatric inpatient population. In fact, studies focusing on specific conditions tend to vary from these general hospitalization patterns. Considering Major Depressive Disorder (MDD) as an example, similar to our study, prior literature has shown increased hospitalization duration for female gender and older patients (Fugger et al., 2020; Savoie et al., 2004). However, being single, unemployed, or one’s living arrangements were not established as significant indicators of hospitalization duration among adult MDD patients (Barnow et al., 1997; Cheng et al., 2022; Ismail et al., 2012). These differences between MDD and BPD patients in terms of socio-demographic predictors of hospitalization duration highlight the need and importance of studying the issue for individual conditions and disorders. As discussed before, we are unaware of any prior studies deliberately focusing on BPD.

The hospitalization index was found to be statistically associated with the hospitalization duration with more advanced hospitalizations being slightly shorter in comparison to earlier ones. While we are unaware of prior evidence to suggest that more advanced hospitalizations are generally shorter for BPD patients, one may speculate that treatment strategies may be better and faster tailored to patients that have already been treated in the same institute, possibly by the same staff, in the past. Similarly, it may be partially explained by BPD symptom severity which, in many cases, tends to decrease over time Videler et al. (2019). These and similar reasons may account for this seemingly mild connection. It is important to note in this context that any hospitalization duration generally agrees with its subsequent one, pointing to a solid temporal pattern. In other words, a short/long hospitalization is generally aligned with the duration of its preceding one, while being slightly shorter.

Importantly, this work has several limitations which offer fruitful avenues for future research. First, our sample is taken from a single mental healthcare center. Nonetheless, official data from the Israeli Ministry of Health (2019) shows that Shalvata is roughly representative of the Israeli mental healthcare system in almost every examined measure (e.g., patient characteristics, hospitalization outcome, etc.) for at least the last decade. In addition, the sample’s socio-demographics distribution generally agrees with prior BPD studies with respect to gender (Links et al., 1988; Sansone & Sansone, 2011), age (Wilkinson-Ryan & Westen, 2000), marital status (Links & Heslegrave, 2000), education level (Gunderson & Kolb, 1978), and living arrangement (Swartz et al., 2011). In a similar manner, for the female patient population, Ryden et al. (2008) reported similar employment status distribution. Moreover, the distribution of the hospitalization duration generally aligns with multiple studies of BPD (Bloom et al., 2012). In Shalvata, as in all public hospitals in Israel, hospitalization duration is primarily determined by the medical staff and the specific patient and their needs. Other factors, such as insurance, are secondary and play a minor part in determining hospitalization duration. As such, we plan to extend our investigation outside of Israel in the future. Second, it is also important to note that global and local circumstances such as political tensions and the COVID-19 pandemic could have influenced the hospitalization patterns of the studied population (Decio et al., 2022; Yaniv-Rosenfeld et al., 2023a). These events are hard to control in our context but we believe that these merit a dedicated future investigation. Third, potential changes in one’s socio-demographics during their hospitalization are not explicitly considered (i.e., an employed patient at intake may turn unemployed during the hospitalization period). Finally, specific diagnostic categories or comorbidity (e.g., substance use), hospitalization circumstances (i.e. crisis hospitalization, out-of-home placement, etc), and individual treatment plans were not considered as part of our analysis. Such an investigation requires more extensive and detailed data, different levels of “grouping” by different diagnosis codes, documentation of hospitalization circumstances, and treatment plans; All these could help reveal more fine-grained differences in BPD patients’ hospitalization duration. We plan to pursue this ambitious task in future studies. Lastly, while our results do point to several socio-demographic features that are significantly associated with one’s hospitalization duration, they need not necessarily translate well to any interpretation of a “successful” hospitalization as the latter is both often subjective and outside the scope of this work (Perkins, 2001; Yaniv-Rosenfeld et al., 2023b). In future studies, we plan to investigate additional indicators of hospitalization success such as the time between consecutive hospitalizations, the prevalence of the revolving door phenomenon among BPD patients, and their adherence to follow-up meetings and pharmacological treatment.

The presented results have both theoretical and clinical potential implications. From a basic research perspective, our study points to gender, age, employment, living arrangement, hospitalization index, and prior hospitalization duration as potentially valuable indicators for modeling and understanding the inpatient treatment dynamics of BPD patients. From a clinical perspective, more suitable treatment plans and hospitalization goals could, and arguably should, be defined based on one’s expected hospitalization duration at admission. This seems to be especially merited for at-risk populations for prolonged hospital duration such as females, young adults, unemployed, and those living with their parents. Since setting goals and a time framework for hospitalization at admission is an essential element in the clinical management of BPD inpatients, the presented results can help clinicians make more informed decisions and shape the existing mental health policy in this regard. We consider this work as a necessary step towards a more ambitious goal: to tailor a suitable and effective hospitalization time framework for a given BPD patient at admission. Regulating the hospitalization duration of BPD patients is of fundamental importance given the intricacy of treating this population and the adverse consequences of unnecessary prolonged hospitalizations of these patients.

## Supplementary Information

Below is the link to the electronic supplementary material.

Supplementary file1 (DOCX 130 kb)

## Data Availability

The data is available upon written request from the authors.
