# Supplementary material for: Socio-demographic Predictors of Hospitalization Duration Among Patients with Borderline Personality Disorder
Source: Adm Policy Ment Health. 2024 May 30;52(5):923–31. doi: 10.1007/s10488-024-01388-w (PMC12449330; doi:10.1007/s10488-024-01388-w)
Supplement: Supplementary file 1 — Supplementary file1 (DOCX 130 kb) [file 10488_2024_1388_MOESM1_ESM.docx]

**Supplementary material: Socio-Demographic Predictors of Hospitalization Duration Among Patients with Borderline Personality Disorder**


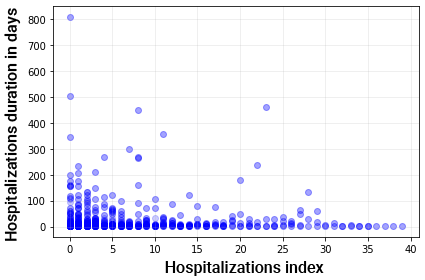


Figure 1: Histogram of the number of hospitalizations per patient in our sample. The histogram consists of 10 bins, each of size 4, thus covering the entire range of 1 to 40 hospitalizations per patient in our sample.


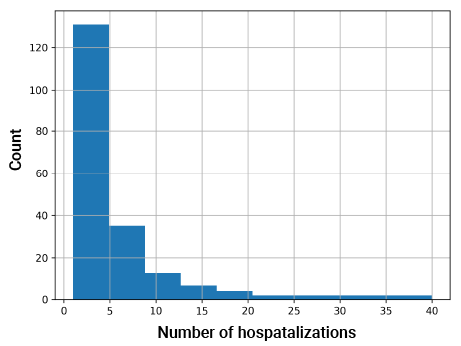


Figure 2: Visit index and the visit's duration. Color intensity denotes the number of samples (the more the darker).


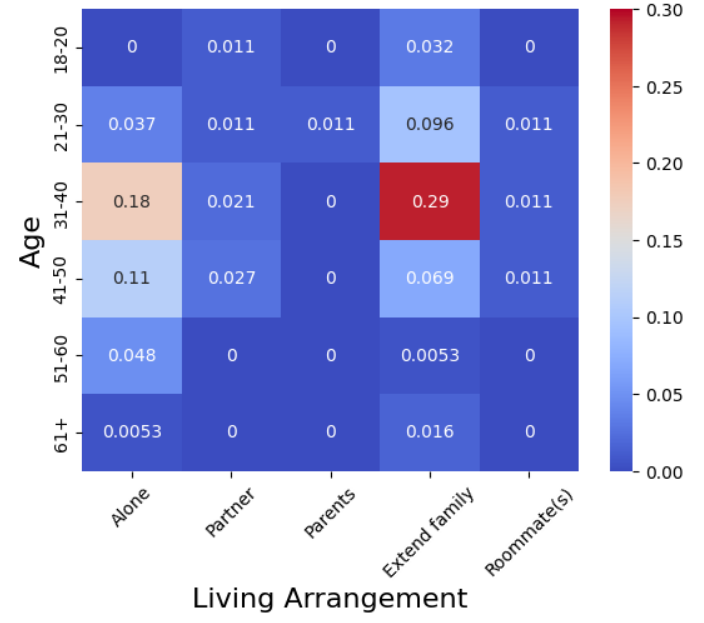


Figure 3: Heatmap of the correlation between age and living arrangement.
